# Associations Between Motor Competence and Mental Health in Youth: A Systematic Review and Meta‐Analysis

**DOI:** 10.1002/ejsc.70220

**Published:** 2026-07-03

**Authors:** Tanja Pöyliö, Anni Pohjanvirta, Katie Robinson, David R. Lubans, Timo Jaakkola, Mikko Huhtiniemi

**Affiliations:** ^1^ Faculty of Sport and Health Sciences University of Jyväskylä Jyväskylä Finland; ^2^ Centre for Active Living and Learning College of Human and Social Futures University of Newcastle Callaghan New South Wales Australia; ^3^ Hunter Medical Research Institute (HMRI) New Lambton New South Wales Australia

**Keywords:** adolescent, children, mental ill‐health, motor skills, psychological health

## Abstract

Motor competence (MC) is essential for an active lifestyle, enabling confident and effective participation in physical activities. Emerging evidence suggests that MC may also benefit young people's mental health. Therefore, this review aimed to synthesize evidence on the association between MC and mental health in youth. We conducted a systematic search of five databases: APA PsycINFO, CINAHL Complete, PubMed, Scopus and SPORTDiscus. Eligible studies were peer‐reviewed, published in English after 2014, and included healthy youth aged 5–19 years. Studies were required to report a quantitative association between at least one aspect of MC and mental health. Screening, data extraction, and risk of bias assessment were performed independently by two researchers. Study quality was evaluated using NHLBI Quality Assessment Tool. We used a structural equation modeling approach for meta‐analysis, and publication bias was assessed using funnel plots and Egger's regression test. We identified 6049 records after removing duplicates. 17 studies met the inclusion criteria, of which two lacked the necessary statistics for meta‐analysis. Most studies were cross‐sectional (*n* = 14) and included children (*n* = 13). We observed a small but significant association between MC and mental health (effect size = 0.30, 95% confidence interval 0.20–0.40). Significant moderators included study design, age, type of MC assessment, risk of bias, domains of MC and mental health. Our findings suggest that MC was associated with mental health in youth. Participants' age, domains of MC and mental health emerged as the most relevant moderators. Further experimental studies, particularly in adolescents, are needed to investigate underlying mechanisms.

**Trial Registration:**

PROSPERO: CRD42024495894

## Highlights

- Motor competence may play a meaningful role in supporting mental health in youth.
- This relationship differs depending on age and measurement characteristics.
- Current evidence is dominated by cross‐sectional studies and younger samples, highlighting the need for more experimental and longitudinal research, especially in adolescents.

## 1 Introduction

Physical inactivity and poor mental health among youth are public health concerns (Farooq et al. 2020; GBD 2019 Mental Disorders Collaborators 2022; Guthold et al. 2020). Globally, only 19% of adolescents aged 11–17 years meet the recommended guidelines of 60 min of daily moderate‐to‐vigorous‐intensity physical activity (MVPA) (Farooq et al. 2020; Guthold et al. 2020; World Health Organization 2020). At the same time, the prevalence of mental ill‐health among young people has risen, a trend that has been further exacerbated by the impacts of the COVID‐19 pandemic (GBD 2019 Mental Disorders Collaborators 2022; Racine et al. 2021; Santomauro et al. 2021; Vos et al. 2020). For example, major depressive disorders have increased by 27.6% across the population, while anxiety disorders have risen by 25.6% since the pandemic's onset (Santomauro et al. 2021). However, mental health is not just the absence of mental ill‐health (World Health Organization 2022). According to the World Health Organization (World Health Organization 2022), it is a “state of mental well‐being that enables people to cope with stresses of life, to realize their abilities, to learn well and work well, and to contribute to their communities”. Mental health includes both positive indicators, such as global self‐esteem, well‐being, and quality of life, as well as negative indicators, such as depression, anxiety, and peer problems (Lubans et al., et al., 2016; World Health Organization 2022). Childhood and adolescence are critical periods for mental health promotion and the prevention of mental ill‐health, as schools provide a unique opportunity to reach nearly all children and adolescents. These developmental stages are characterized by rapid physical, cognitive, and social changes, including puberty, brain maturation (Lebel and Deoni 2018), and evolving social relationships and behaviors across family, peer, and educational context (Viner et al. 2012). The transition from childhood to adolescence is considered a particularly vulnerable period, during which many mental disorders first emerge (Patel et al. 2007; Solmi et al. 2022), highlighting the importance of investigating factors that contribute to mental health.

Engaging in physical activity (PA) is an important lifestyle behavior that improves mental health and well‐being in young people (Biddle et al. 2019; Rodriguez‐Ayllon et al. 2019; Teychenne et al. 2025; White et al. 2024). Developing motor competence (MC) is essential for an active lifestyle, as it underpins the ability to engage confidently and effectively in a wide range of PA (Barnett et al. 2016, 2022; Hulteen et al. 2018). MC is an umbrella term to describe a person's skills and movement patterns needed to execute goal‐directed movements (Goodway et al. 2021; Robinson et al. 2015). It includes locomotor, object control and stability skills (Goodway et al. 2021), which play a crucial role in children's physical and psychosocial growth and development, enabling them to actively explore and navigate their environment (Adolf and Hoch 2019). Stodden et al. (2008) developed a model emphasizing the importance of MC in promoting PA and healthy body weight in youth. In addition, to provide a foundation for participation in a range of PA, MC is suggested to contribute mental health benefits for young people (Hill et al. 2023; Lima et al. 2022).

The relationship between PA and mental health may be explained by a complex interplay of neurobiological and psychosocial factors (Kandola et al. 2019; Lubans et al. 2016; White et al. 2024). Similarly, MC may influence mental health through both direct and indirect pathways, although the underlying mechanisms are not fully understood (Gu et al. 2018, 2019; Hill et al. 2023; Mancini et al. 2018a, 2018b). For instance, Gu et al. (2018) demonstrated that among preschool‐aged children in the United States, MC was associated with mental health both directly and indirectly through MVPA. Previous reviews have investigated the links between MC and outcomes related to mental health, particularly social‐emotional and psychosocial characteristics. For example, Hill et al. (2023) reviewed 12 studies, most of which involved preschool‐aged children, and found preliminary evidence supporting a pathway between MC and social‐emotional development. Their review focused on outcomes such as self‐regulation, life skills, and social skills, and due to substantial heterogeneity among the included studies, they were unable to quantitatively synthesize the evidence. Similarly, Burton et al. (2023) reported positive associations between MC and psychosocial characteristics; however, their review included constructs such as self‐efficacy/confidence, perceived MC, and motivation.

While these reviews provide valuable insight into the social‐emotional and psychosocial outcomes associated with MC, no previous review or meta‐analysis has quantitively synthesized evidence on the association between MC and global mental health outcomes in children and adolescents. Given the growing recognition that mental health encompasses both the absence of mental ill‐health and the presence of positive mental health (World Health Organization 2022), a broader examination of this relationship was warranted. Therefore, the primary aim of our review was to quantitatively synthesize cross‐sectional, longitudinal, and experimental studies examining associations between MC and mental health outcomes in youth. Our secondary aim was to explore potential moderators of this association, including age, sex/gender, study design, risk of bias, type of MC assessment, and the domains of MC and mental health.

## 2 Methods

Our systematic review and meta‐analysis were conducted according to the Preferred Reporting Items for Systematic Reviews and Meta‐Analysis (PRISMA) statement and following Prisma in Exercise, Rehabilitation, Sport medicine and SporTs (PERSiST) guidance (Ardern et al. 2022; Page et al. 2021). Our protocol was prospectively registered with the international Prospective Register of Systematic Reviews (PROSPERO) database on the 1st of January 2024 (CRD42024495894).

### 2.1 Search Strategy and Eligibility Criteria

We performed a structured literature search using the following databases: APA PsycINFO, CINAHL Complete, PubMed, Scopus and SPORTDiscus. The database search included published and peer‐reviewed articles in English, from 1st of January 2014 to 14th of May 2025 to capture the most recent evidence in rapidly changing developmental and social context. In addition, searches of reference lists from included articles were performed to identify any further publications and a subject matter expert was contacted for further identification of studies (Muka et al. 2020). The final search terms included keywords relating to (i) age, (ii) MC, and (iii) global mental health (Supporting Information S1: Table 1).

To be eligible for inclusion, all studies needed to fulfill the following criteria of the Population (1), Intervention (2), Comparator (3), and Outcome (4) (PICO) framework (McKenzie et al. 2024). (1) Participants: typically developing school‐aged children and adolescents (5–19‐years‐old), including those with overweight or obesity (Sawyer et al. 2018); (2) Intervention/exposure characteristics: studies that provided a quantitative assessment of the association between MC and global mental health; (3) Comparison: cross‐sectional, longitudinal and experimental studies were eligible for inclusion; (4) Outcomes: MC assessed using a product, process, or combined methods. It needed to include fundamental movement skills, motor coordination, or other goal‐directed human movement as described in earlier reviews (Barnett et al. 2016; Hill et al. 2023). If fine motor skills were analyzed in a summary score with gross MC, the study was eligible for inclusion. Studies also needed to include a measure of global mental health. As noted by the World Health Organization (2022), mental health is not just the absence of mental ill‐health but also includes positive indicators. Therefore, we included positive aspects of mental health such as mental well‐being, self‐esteem, and health‐related quality of life, and mental ill‐health measures, such as mental health disorders (e.g., symptoms of depression and anxiety) and preclinical mental health problems (e.g., perceived stress). Measurements related only to self‐perceptions (e.g., body image) and social well‐being were not included in the meta‐analysis.

### 2.2 Selection Processes

We used Covidence Software for the exclusion of duplicate studies, titles and abstract screening, and full text reading. In the first phase, two researchers independently screened titles and abstracts to ensure eligibility. In the second phase, the relevant full texts were extracted and independently reviewed by the same two researchers. Possible disagreements were discussed and decided together with the assistance of a third researcher when needed.

### 2.3 Data Extraction

Data from eligible studies were extracted by two researchers using a standardized extraction form. The following data were extracted from the included studies: study background (first author's name, year of publication, study title, and location), sample characteristics (sample size, sex/gender, and mean age), research design and duration, and assessment details for MC and mental health (outcome and instrument, and association between outcomes [strength]). If some relevant information was missing, the corresponding author was contacted.

### 2.4 Study Risk of Bias Assessment

Two researchers independently assessed the methodological quality of each eligible study using the National Heart, Lung, and Blood Institute (NHLBI) ‘Quality Assessment Tool for Observational Cohort and Cross‐sectional Studies’ (National Heart et al. 2021). Any disagreements were discussed and solved in subsequent meetings. A third researcher was used to guide the overall risk of bias assessment of the studies.

### 2.5 Effect Measures

Summary measures included standardized regression coefficients and correlation coefficients. In the studies that utilized multiple assessments of MC or global mental health, individual test scores were extracted and analyzed individually. All results were converted to Cohen's *d* effect size (ES) which are defined as small (0.20), medium (0.50), and large (0.80) (Cohen 1988). For the meta‐analysis, all mental ill‐health ES were converted to align with the positive mental health direction.

We used a structural equation modeling approach to multilevel meta‐analysis. This approach does not limit the assumption of independence, and multiple ES can be included from each study (Cheung 2023). We used restricted Maximum Likelihood to calculate *τ* ^2^ values for the variance of the distribution of true ES (Harrer et al. 2021; Viechtbauer 2005). For every pooled ES, confidence intervals (CI) based on 95% likelihood were computed. All analyses were conducted using the metaSEM package (Cheung 2023) in RStudio 4.4. (Team 2025).

We report on the *I* ^2^ statistics as a measure of variability in the observed ES (i.e., heterogeneity) (Higgins et al. 2003). An *I* ^2^ value ranging from 0% to 40% may indicate negligible heterogeneity, 30%–60% moderate heterogeneity, 50%–90% substantial heterogeneity, and 75%–100% considerable heterogeneity (Deeks et al. 2024). Publication bias was analyzed using funnel plots (Page et al. 2024) and Egger's regression asymmetry tests (Egger et al. 1997). The sensitivity analyses were conducted using the trim‐and‐fill and the leave‐one‐out procedures. Th trim‐and‐fill method estimates the potential impact of publication bias by imputing missing studies to restore funnel plot symmetry and recalculating the pooled effect size (Duval and Tweedie 2000). Leave‐one‐out analyses were performed by sequentially excluding each study to assess whether the pooled effect size was unduly influenced by any individual study. Moderator analyses were conducted to determine whether the association between MC and mental health differed according to age (children and adolescents), sex/gender (girls and boys), study design (cross‐sectional and longitudinal), risk of bias (low, moderate and high), type of MC assessment (product‐ or process‐oriented or combination), the domain of MC (total sum score of gross MC, combined fine and gross MC, object control and locomotor skills) or mental health outcome type (global self‐esteem, well‐being, health‐related quality of life, internalizing problems, externalizing problems, and total difficulties). The *R* ^2^ values were calculated to assess the explained variance for each potential moderator variable (Harrer et al. 2021). For interpreting the moderating effects, we used following guidelines: very weak (*R* ^2^ < 0.02), weak (0.02 < *R* ^2^ < 0.13), moderate (0.13 < *R* ^2^ < 0.26), substantial (*R* ^2^ > 0.26) (Cohen 1988).

## 3 Results

### 3.1 Study Selection

**FIGURE 1:**
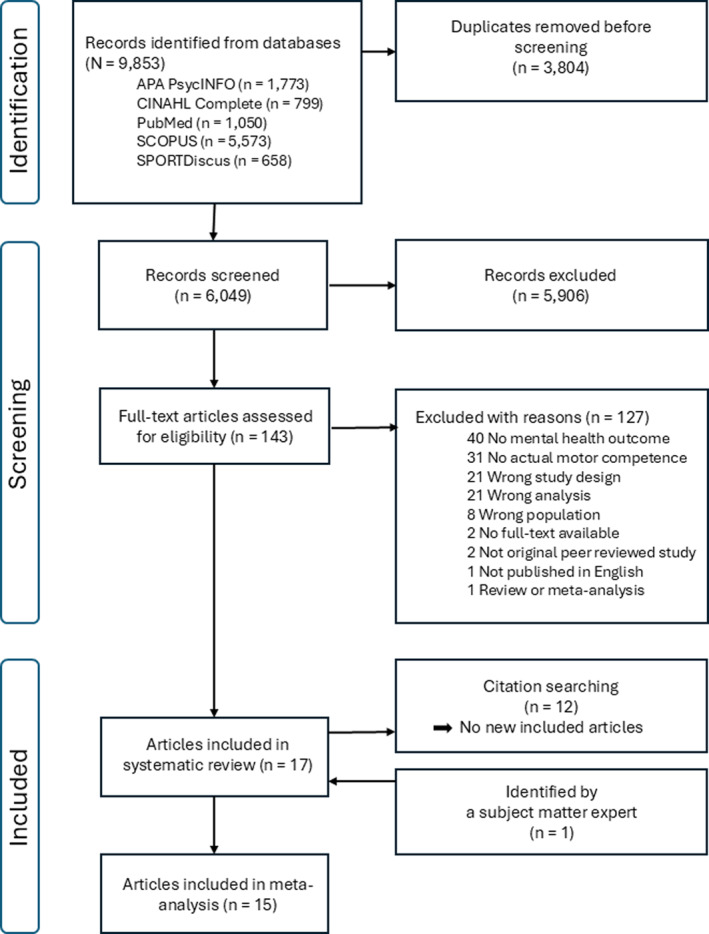
PRISMA flow diagram for study selection process.

The PRISMA flow diagram (Figure 1) outlines the study selection process. A total of 9853 potentially relevant articles were identified from databases. After removing duplicates, 6049 articles were screened based on their titles and abstracts. Altogether, 5906 articles were excluded during the screening phase, and 143 records were assessed for eligibility via full‐text reading. 16 articles met the full inclusion criteria, and citation searching yielded no additional studies. However, one additional article was identified by a subject matter expert, bringing the total to 17 articles included in the systematic review. Of these, two were excluded from the meta‐analysis due to the unavailability of necessary supplementary data from the authors. Consequently, 15 articles were included in the meta‐analysis.

### 3.2 Overview of Studies

We provide a detailed description of the studies included in Supporting Information S1: Table 2. The reviewed studies were conducted across various countries. Five studies were from Australia, and two from the USA and China. Additionally, there were eight countries that each contributed one study (Belgium, Brazil, Canada, Great Britain, Netherlands, Portugal, Spain, and Switzerland). The study design primarily consisted of cross‐sectional studies (*n* = 14) and three longitudinal studies. No experimental studies were found. Our systematic review included a total of 6304 participants with a mean age range from 5.3 to 14.21. The participant samples were mostly children (5–11 years, *n* = 13) and the sample size range varied from 55 to 1088.

MC was operationalized as locomotor skills (*n* = 2), object control skills (*n* = 5) and total sum scores of either gross MC (*n* = 8) or combined fine and gross MC (*n* = 6). MC was examined using product‐ and process‐oriented and combined measurements. The Movement Assessment Battery for Children 2 (MABC‐2) was the most used product‐oriented measure (de Medeiros et al. 2022; Li et al. 2025; Mancini et al. 2016, 2018a; Noordstar and Volman 2020; Redondo‐Tebar et al. 2021) while PE Metrics was the most applied process‐oriented measurement (Chen et al. 2021; Gu et al. 2018, 2019). The Canadian Agility and Movement Skill Assessment (CAMSA) was the only measure to utilize both process‐ and product‐orientation (Sortwell et al. 2024). None of the studies measured solely stability skills.

Mental health was operationalized as global self‐worth or self‐esteem (*n* = 8), emotional, psychological or psychosocial well‐being (*n* = 4), health‐related quality of life (*n* = 3), internalizing problems such as anxiety or depression (*n* = 6), externalizing problems such as peer problems or hyperactivity (*n* = 3), and total difficulties (*n* = 2). Many studies utilized multiple measurement tools and aspects of global mental health. The commonly used tools were Strength and Difficulties Questionnaire (SDQ) (de Medeiros et al. 2022; Mancini et al. 2018a, 2018b; Tang et al. 2023) and the global self‐worth subscale from the Self‐Perception Profile for Children (SPPC) (Bardid et al. 2016; Lalor and Brown 2016; Mancini, Rigoli, Roberts, Heritage, and Piek 2018a; Noordstar and Volman 2020; Sortwell et al. 2024).

### 3.3 Risk of Bias

The risk of bias within studies is presented in Supporting Information S1: Table 3. All studies were categorized based on their level of concern: low (*n* = 10), moderate (*n* = 6), and high (*n* = 1). All studies clearly stated their research questions, and nearly all (94%) used reliable instruments for MC, and all used reliable instruments for mental health. Only two studies failed to define the population, as they did not specify the area or the schools from which the population was drawn.

**FIGURE 2:**
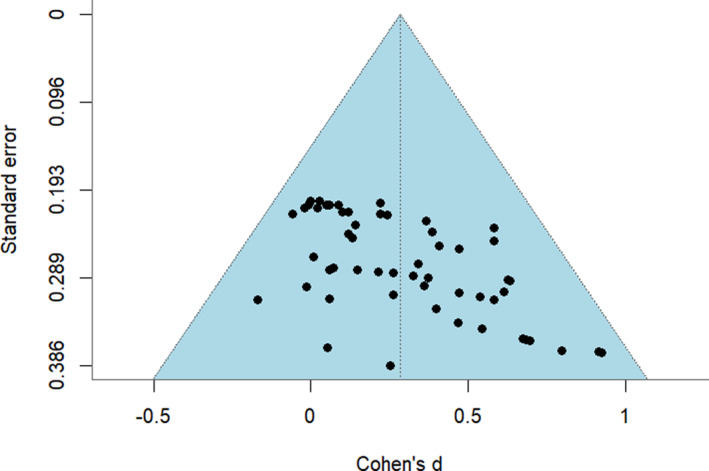
Funnel plot of Cohen's *d* effect sizes plotted against their standard errors.

We used a funnel plot of Cohen's *d* ES against their standard errors to visually represent potential publication bias (Figure 2). The plot showed slight asymmetry (Egger et al. 1997). This was supported by a significant Egger's regression test (*z* = 4.4421, *p* < 0.001). The trim‐and‐fill sensitivity analysis did not impute any missing studies, and the pooled ES remained unchanged after adjustment. Leave‐one‐out sensitivity analyses indicated minimal variation in the pooled ES (range: 0.28–0.30), with no changes in the direction or statistical significance of the effect.

### 3.4 Synthesis of Meta‐Analysis Results

#### 3.4.1 Overall Associations

We observed a small positive association between MC and global mental health (ES = 0.30, 95% CI 0.20–0.40) (Table 1). For this effect, there was moderate heterogeneity both within (*I* ^2^ = 0.53) and between (*I* ^2^ = 0.41) studies.

**TABLE 1:** Associations between motor competence and mental health in school‐aged youth.

Variable	k	Number of ES	ES (Cohen's *d*)	Lower 95% CI	Upper 95% CI	*I* ^2^_2	*I* ^2^_3	*τ* ^2^_2	*τ* ^2^_3	*R* ^2^_2	*R* ^2^_3
Overall association	15	56	0.30	0.20	0.40	0.526	0.408	0.031	0.024		
Moderator analyses											
Age category								0.031	0.016	0.000	0.351
Children (5–11 yrs)	12	45	**0.25**	0.16	0.35						
Adolescents (12–19 yrs)	2	9	**0.50**	0.28	0.72						
Both	1	2	0.36	−0.01	0.73						
Sex/gender								0.031	0.105	0.000	0.000
Boys	5	11	0.14	−0.18	0.45						
Girls	5	11	0.08	−0.23	0.39						
Study design								0.031	0.022	0.003	0.110
Cross‐sectional	13	49	**0.27**	0.17	0.39						
Longitudinal	3	7	**0.41**	0.18	0.63						
Risk of bias								0.031	0.021	0.022	0.124
Poor (high risk of bias)	1	2	0.03	−0.36	0.42						
Fair (Moderate risk of bias)	5	10	**0.28**	0.10	0.46						
Good (Low risk of bias)	9	44	**0.33**	0.21	0.45						
Type of MC assessment								0.031	0.024	0.003	0.033
Product‐oriented	9	41	**0.31**	0.18	0.43						
Process‐oriented	5	13	**0.30**	0.13	0.47						
Combination	1	2	0.15	−0.29	0.58						
Domain of MC								0.032	0.021	0.000	0.130
Total sum score of gross MC	8	22	**0.29**	0.15	0.43						
Combined fine and gross MC	4	19	**0.35**	0.17	0.53						
Locomotor skills	2	3	0.24	−0.06	0.54						
Object control skills	5	12	**0.27**	0.12	0.43						
Mental health outcome								0.027	0.005	0.136	0.801
Global self‐esteem	7	13	**0.15**	0.04	0.27						
Well‐being	4	10	**0.23**	0.10	0.36						
Health‐related quality of life	2	9	**0.18**	0.01	0.36						
Internalizing problems	5	20	**0.50**	0.40	0.61						
Externalizing problems	2	2	0.19	−0.07	0.46						
Total difficulties	2	2	**0.48**	0.23	0.74						

*Note:* Results are presented in Cohen's *d* and statistically significant results are bolded. A Cohen's *d* of 0.2 is interpreted as small, 0.5 represents medium and 0.8 a large ES. *I* ^2^_2 = heterogeneity at Level 2 (i.e., between ESs from the same study); *I* ^2^_3 = heterogeneity at Level 3 (i.e., between studies). *τ* ^2^_2 = within study variance; *τ* ^2^_3 = between study variance; *R* ^2^_2 = variance explained at Level 2 (i.e., between ESs from the same study); *R* ^2^_3 = variance explained at Level 3 (i.e., between studies).

#### 3.4.2 Moderators of Effects

Age significantly moderated the association between MC on mental health (*R* ^2^ = 0.35), with stronger ES observed in adolescents (ES = 0.50, 95% CI 0.28–0.72) compared to children (ES = 0.25, 95% CI 0.16–0.35). In contrast, sex/gender did not moderate the relationship (*R* ^2^ = 0.00).

Study design explained a small portion of the heterogeneity found between studies (*R* ^2^ = 0.11). Positive associations were found in both longitudinal (ES = 0.41, 95% CI 0.18–0.63) and cross‐sectional studies (ES = 0.27, 95% CI 0.17–0.39). Similarly, the risk of bias accounted for a small portion of the heterogeneity between studies (*R* ^2^ = 0.12). Positive associations were observed in studies with moderate risk of bias (ES = 0.28, 95% CI 0.10–0.46), and low risk of bias (ES = 0.33, 95% CI 0.21–0.45).

Type of MC assessment explained only a negligible portion of the between‐study heterogeneity (*R* ^2^ = 0.03). Both product‐oriented (ES = 0.31, 95% CI 0.18–0.43), and process‐oriented assessments (ES = 0.30, 95% CI 0.13–0.47) showed significant positive associations with mental health.

Domains of MC explained a small portion of the between‐study heterogeneity (*R* ^2^ = 0.13). Positive associations were found from total sum score of gross MC (ES = 0.29, 95% CI 0.15–0.43), combined fine and gross MC (ES = 0.35, 95% CI 0.17–0.53), and object control skills (ES = 0.27, 95% CI 0.12–0.43). Stability skills could not be analyzed separately, as none of the articles included categorized them independently.

When considering types of mental health outcomes, they explained a substantial proportion of between‐study heterogeneity (*R* ^2^ = 0.80) and a small proportion within study heterogeneity (*R* ^2^ = 0.14). MC was positively associated with global self‐esteem (ES = 0.15, 95% CI 0.04–0.27), well‐being (ES = 0.23, 95% CI 0.10–0.36), health‐related quality of life (ES = 0.18, 95% CI 0.01–0.36), internalizing problems (ES = 0.50, 95% CI 0.40–0.61), and total difficulties (ES = 0.48, 95% CI 0.23–0.74). No significant association was found for externalizing problems (ES = 0.19, 95% CI −0.07–0.46).

## 4 Discussion

### 4.1 Summary of Findings

This is the first systematic review and meta‐analysis to synthesize studies examining the associations between MC and global mental health in youth. Our findings indicate a small positive association (ES = 0.30, 95% CI 0.20–0.40) between MC and global mental health, consistent across both cross‐sectional and longitudinal studies. Meta‐regression analysis revealed several factors that explained the heterogeneity in ES. Associations were stronger in adolescent populations, for overall MC, internalizing problems, and total difficulties compared with children, object control or locomotor skills and other types of mental health outcomes. Therefore, our study highlights the importance of MC for mental health and provides valuable insights into the moderating factors.

### 4.2 Motor Competence and Mental Health

Our review provides a quantitative synthesis of original studies examining the associations between MC and global mental health through the adoption of a broader conceptualization of mental health. Recognizing that mental health exists on a continuum and is not defined solely by the presence or absence of mental disorders (World Health Organization 2022), we included a wide range of mental health outcomes, such as depression, anxiety, global self‐esteem, and psychological well‐being. Our findings are consistent with earlier related reviews of MC. Burton et al. (2023) reported positive correlations between MC and psychosocial characteristics (*r* = 0.07–0.34), while Hill et al. (2023) identified consistent associations between MC and social‐emotional development. However, Burton et al. (2023) included studies examining physical activity‐related self‐efficacies, perceived competencies, and motivational aspects, whereas Hill et al. (2023) emphasized social‐emotional outcomes such as social behavior, self‐regulation, and motivational climate, rather than broader mental health dimensions. Notably, only one study included in Hill et al. (2023) overlapped with the present review, highlighting the distinct evidence base of the two reviews. Although, the overall ES observed in our meta‐analysis was small, the association between MC and mental health was consistently evident across different MC domains and mental health outcomes. These findings extend previous evidence linking MC to psychosocial and social‐emotional outcomes by demonstrating that MC is also associated with broader dimensions of mental health in children and adolescents.

We used meta‐regression to identify factors that may explain the high levels of heterogeneity in our results and identified several significant moderators, including age, sex/gender, study design, risk of bias, type of MC assessment, and domains of MC and mental health outcomes. These moderators explained a notable portion of the variance, particularly between studies. Firstly, participant age moderated the association between MC and mental health, with adolescence being a stronger moderator than childhood. Adolescence is a period of developmental changes, including biological development, school transitions, and changes in peer and parental relationships, which can protect or undermine mental health in youth (Patel et al. 2007; Solmi et al. 2022). This finding is consistent with the review of Cadenas‐Sanchez et al. (2021), where they found higher results in adolescents in the context of physical fitness and mental health compared to children. As noted by Utesch et al. (2019), physical fitness and MC are moderately associated, with this relationship strengthening from childhood through adolescence. However, this finding should be interpreted with caution, as it is derived from a small number of adolescent studies, potentially limiting the reliability of the estimate. In contrast, sex/gender was not a significant moderator, and it did not explain the variance within studies in our results. This finding is intriguing, as boys typically possess better object control skills and fewer internalizing problems than girls (Barnett et al. 2016; GBD 2019 Mental Disorders Collaborators 2022; Santomauro et al. 2021), but these differences were not observed in our study. Similarly, in the review of Cadenas‐Sanchez et al. (2021), sex/gender was not a significant moderator. Many of the included studies did not perform separate analysis for boys, girls or others, resulting in only 10 studies being included in this meta‐regression and only boys/girls' categories were able to be established. We combined positive and negative mental health indicators, which may have influenced the moderation of sex/gender. Overall, the existing evidence supports that MC has benefits for mental health in youth, regardless of their age or sex/gender.

The included studies utilized product‐oriented and process‐oriented measurements, with one study using a combination of both. Product‐oriented instruments evaluate outcomes, whereas process‐oriented instruments examine the quality of movement (Bardid et al. 2019; Logan et al. 2017). Due to the widespread use of the MABC‐2 total score, fine motor skills were included in the total scores alongside gross motor skills in many studies. This combined score of fine and gross MC was the highest moderator among the domains of MC, indicating that fine motor skills might play a role in the relationship between MC and global mental health. However, when these competencies were examined separately, gross MC was more strongly associated with total difficulties than fine MC (Li et al. 2025). Additionally, Gu et al. (2018) found small and significant correlations between locomotor skills, object control skills and total MC with psychosocial functioning. It is important to note that in our review, both process‐ and product‐oriented assessments and all domains of MC showed similar effects. Our findings underscore the importance of considering what type of measurement tool is used and the role of fine and gross motor skills in understanding the relationship between MC and mental health.

Mental health outcomes explained the largest amount of heterogeneity in all the meta‐regressions. The results revealed small positive associations for global self‐esteem, well‐being, and health‐related quality of life and stronger associations for internalizing problems and total difficulties. Externalizing problems was the only outcome that was not statistically significant. In our study, the association from MC to negative indicators of mental health is higher than those to positive indicators. Our results highlight the need for multi‐method assessments of mental health, including both positive and negative indicators and experimental study designs to clarify these associations.

Funnel plot asymmetry may indicate publication bias, small‐study effects or heterogeneity (Egger et al. 1997; Sterne et al. 2011). In this study, both the funnel plot and Egger's test indicated asymmetry, suggesting the presence of small‐study effects or heterogeneity. However, a trim‐and‐fill analysis did not impute missing studies, and the pooled ES remained unchanged, indicating that the observed asymmetry is unlikely to be driven by missing, potentially unpublished, studies (Duval and Tweedie 2000). In addition, leave‐one‐out analyses further showed stable results, supporting the robustness of the findings. Overall, while some evidence of asymmetry was observed, the sensitivity analyses suggest that the main conclusions are robust.

It is hypothesized that MC may have direct or indirect effects on mental health (Gu et al. 2019; Hill et al. 2023; Lopes et al. 2022; Mancini, Rigoli, Roberts, Heritage, and Piek 2018b), and indirect associations have gained more support. These effects may be influenced by factors such as social domains (e.g., peer relationships), physical fitness (e.g., aerobic fitness) or perceived MC (Bardid et al. 2016; Gu et al. 2019; Hill et al. 2023; Mancini, Rigoli, Roberts, Heritage, and Piek 2018b). For example, some included studies reported that MC had indirect effects on mental ill‐health through peer problems (Mancini, Rigoli, Roberts, Heritage, and Piek 2018b) and on mental health through health‐related physical fitness (Gu et al. 2019). Additionally, Bardid et al. (2016) indicated that low perceived MC can negatively impact children's global self‐worth despite high levels of actual MC. These results support indirect associations through various psychosocial or neurobiological mechanisms, as described in reviews by Hill et al. (2023) and Lubans et al. (2016). Psychosocial mechanisms are based on theoretical frameworks that propose satisfying basic psychological needs as key to achieving well‐being (Lubans et al. 2016; Ryan and Deci 2002; Ryff and Keyes 1995), while neurobiological mechanisms involve different structural and functional compositions of the brain, such as inflammation or oxidative stress (Kandola et al. 2019; Lubans et al. 2016). Our findings underscore the multifaceted ways in which MC can positively influence mental health in youth.

### 4.3 Strengths and Limitations

A significant strength of our meta‐analysis is the use of multilevel structural equation modeling, consistent with best practices in meta‐analytic research in the field (Kadlec et al. 2023). However, several limitations should be considered. First, both MC and mental health were assessed using a variety of tools differing in validity and reliability, which may have contributed to between‐study heterogeneity. While our broad conceptualization enhances coverage, it also introduces variability across studies.

Second, high heterogeneity was observed across studies. This may reduce the precision of the pooled estimate and suggests that the magnitude of the association may vary across populations and study contexts. Although funnel plot inspection and Egger's regression test indicated asymmetry, the trim‐and‐fill procedure did not impute potentially missing studies, and the pooled effect size remained the same. In addition, leave‐one‐out analyses demonstrated that pooled ES was not influenced by any single study. Thus, while small‐study effects cannot be ruled out, there is no clear evidence that publication bias substantially influenced the pooled results.

Third, nearly all studies included in the meta‐analysis were observational, which limits our ability to interpret causality. Only a few longitudinal studies suggested a potential relationship where MC influenced mental health. Fourth, as most studies focused on children, the findings may be less generalizable to adolescents, and the moderating effect of age should therefore be interpreted with caution. Furthermore, the moderating effect of total difficulties was based on only two ES and should likewise be interpreted cautiously.

### 4.4 Future Directions

Our systematic review and meta‐analysis included only 17 studies, suggesting the need for further research to examine the associations between MC and global mental health in youth. Specifically, we were unable to find any experimental studies and only three longitudinal studies. Therefore, new experimental and longitudinal research is necessary to investigate causality and explore the underlying mechanisms that might explain the effects of MC on mental health. For instance, investigating the impact of perceived MC is warranted, since there is some evidence of the associations between MC and perceived MC on mental health (Bardid et al. 2016; Burton et al. 2023). Only two studies involved adolescents aged 12–19 years. Therefore, more research is needed on adolescents to provide a better understanding of how MC and mental health are interrelated and whether these relationships differ across developmental stages.

## 5 Conclusions

In conclusion, our findings indicate a small but significant positive association between MC and global mental health in children and adolescents, with age, domains of MC and mental health outcomes being the most relevant moderators. Our results support the hypothesis that good MC may enhance mental health in youth. However, the included studies featured only a small number of longitudinal studies and lack of experimental studies, highlighting the need for high‐quality experimental research. It is important to note that not all PA interventions improve mental health (Vella et al. 2023), and future research should consider using evidence‐based strategies (e.g., (Lubans et al. 2017)).

## Author Contributions

T.P. was responsible for the study's conceptualization and design, data collection, analysis, initial manuscript drafting, and subsequent revisions. A.P. handled study design, data collection and reviewed the manuscript. K.R. supplied the data collection instruments, supervised the analyses and reviewed the manuscript. D.R.L. provided methodological expertise, contributed to the study design and conceptualization, and reviewed and revised the manuscript. T.J. contributed to the study design and conceptualization and reviewed and revised the manuscript. M.H. was involved in the study design and conceptualization and reviewed and revised the manuscript. All authors approved the final version of the manuscript.

## Funding

This work was supported by the SchoolWell‐project, funded by the Strategic Research Council within the Academy of Finland [Grant 352512], and the Finnish Ministry of Education and Culture [Grant OKM/70/626/2017].

## Ethics Statement

The authors have nothing to report.

## Conflicts of Interest

The authors declare no conflicts of interest.

## Supporting information


Supporting Information S1

## Data Availability

The data that support the findings of this study are available from the corresponding author upon reasonable request.
