# Supplementary material for: Associations Between Motor Competence and Mental Health in Youth: A Systematic Review and Meta‐Analysis
Source: Eur J Sport Sci. 2026 Jul 3;26(8):e70220. doi: 10.1002/ejsc.70220 (PMC13331750; doi:10.1002/ejsc.70220)
Supplement: Supplementary file 1 — Supporting Information S1 [file EJSC-26-e70220-s001.docx]

Associations between motor competence and mental health in youth: A systematic review and meta-analysis

**Supplementary Online Content**

**Supplementary table 1** Full search strategy

**Supplementary table 2** Key characteristics of studies examining the association between motor competence and global mental health

**Supplementary table 3** Risk of bias assessment checklist for studies (NHLBI Study Quality Assessment Tool for Observational Cohort and Cross-Sectional Studies**)**

**Supplementary table 1** Full search strategy

| Keywords | Search terms |
| --- | --- |
| Population | *“child*” OR “primary school” OR “elementary school” OR “secondary school” OR “secondary education” OR “secondary college” OR “adolescen*” OR “student” OR “teen*” OR “young*” OR “youth” OR “junior high” OR “high school” OR “senior high” Or “young people”* |
| Motor competence | *“actual competen*” OR “athletic competen*” OR “athletic skill” OR “basic movement” OR “motor coordination” OR “foundational movement” OR “functional movement” OR “fundamental motor” OR “fundamental movement” OR “gross motor” OR “locomotor skill” OR “manipulative skill” OR “motor abilit*” OR “motor activit*” OR “motor behaviour” OR “motor behavior” OR “motor competen*” OR “motor coordination” OR “motor development” OR “motor fitness” OR “motor function*” OR “motor learning” OR “motor perform*” OR “motor proficien*” OR “motor skill” OR “movement assessment” OR “movement competen*” OR “movement skill” OR “movement pattern” OR “object control” OR “object manipulation” OR “physical competenc*” OR “skill proficienc*” OR “stabilit* skill”* |
| Global mental health | *”affective disorder” OR “anxiet*” OR “depress*” OR “emotional regulat*” OR “externalizing“ OR “externalising“ OR “fatiq*” OR “happiness” OR “internalizing” OR “internalising” OR “mental condition” OR “mental disorder” OR “mental difficult*” OR “mental functioning” OR “mental health” OR “mental illness” OR “mental ill-health” OR “mental problem” OR “wellbeing” OR “well-being” OR “negative affect” OR “positive affect” OR “psychological disorder” OR “psychological illness” OR “psychological ill-health” OR “psychological stress” OR “satisfaction with life” OR “somatic symptom” OR “mood” OR “optimism” OR “resilience” OR “stress” OR “ self concept” OR “self-concept” OR “self efficacy” OR “self-efficacy” OR “self-assurance” OR “self-confidence” OR “self-esteem” OR “self-image” OR “self-perception”* |
| Where possible the additional filter *human* was used. | |

**Supplementary table 2** Key characteristics of studies examining the association between motor competence and global mental health

| **Author (year)**  **Country** | **Study design** | **Sample** | **MC measure** | **MC grouping** | **MH measure** | **MH grouping** | **Results** |
| --- | --- | --- | --- | --- | --- | --- | --- |
| Bardid (2016)  Belgium | Cross-sectional | N = 161  n = 96 (f)  n = 65 (m)  Mean age = 8.82 ± 0.66 | KTK | Total sum score of gross motor competence | SPPC, Dutch version: global self-worth subscale | Global self-esteem | Correlation coefficient: There weren’t significant correlations found between actual motor competence and global self-worth (r=0.03, p>0.05). |
| Bretz (2022)  Switzerland | Cross-sectional | N = 1823  Preschool n = 459 (f)  n = 484 (m)  School n = 430 (f)  n = 450 (m)  Mean age preschool 5.7 ± 0.57 and school 7.5 ± 0.58 | MOBAK-KG and MOBAK-1-2 | Total sum score of gross motor competence and object control skills | The KIDSCREEN-10 | Health-related quality of life (HRQoL) | Structural equation model: Regarding general HRQoL, there weren’t any associations with basic motor competencies for either boys or girls in preschool. For primary school, significant relationships with general HRQoL, as assessed by the parents, were only found for self-movement among girls (r=0.18, p=0.007). |
| Chen (2021)  China | Cross-sectional | N = 291  n = 125 (f)  n = 166 (m)  Mean age = 9.77 ± 0.58 | PE Metrics: soccer dribbling, passing, and receiving skills | Object control skills | WEMWBS | Well-being | Standardized regression coefficients: The soccer skills test was not significant individual contributor to predicting the psychological well-being for boys (β=0.04) or for girls (β=-0.01). |
| de Medeiros (2022) Brazil * | Cross-sectional | N = 431  n = 240 (f)  n = 191 (m)  Mean age = 8.97 ± 1.03 | MABC-2 | Total sum score of fine and gross motor competence | SDQ-P and PedsQL: psychosocial health subscale | Internalizing problems, externalizing problems, and psychosocial health | The multiple mediator model: Motor proficiency was not directly related to internalizing problems (B=0.00). In the model the path from motor proficiency to externalizing problems (B= -0.10) was significant and it was not significant to psychosocial health (B=0.25)  The information needed for calculating standardized β-value was missing. |
| Gu (2018)  USA | Longitudinal (One academic year) | N = 141  n = 69 (f)  n = 72 (m)  Mean age = 5.37 ± 0.48 | PE Metrics | Total sum score of gross motor competence, locomotor skills, and object control skills | PedsQL 4.0: psychosocial functioning subscale | Well-being | Pearson’s bivariate correlation coefficient: There were significant correlations from locomotor skills (r=0.23), manipulative skills (r=0.26) and motor competence index (r=0.28) to psychosocial functioning (p<0.01). |
| Gu (2019)  USA | Longitudinal (One academic year) | N = 279  n = 148 (f)  n = 131 (m)  Mean age = 12.49 ± 0.89 | PE Metrics: soccer, volleyball, and ultimate frisbee | Object control skills | CES-DC and PedsQL 4.0 | Health-related quality of life, depression, well-being | Bivariate correlation: Both depression (r= -0.23) and HRQOL (r=0.20) were significantly correlated to overall motor competence (p<0.01).  Structural equation model: Path coefficient from motor competence to mental health outcomes (β=0.22, p=0.29) were not significant in this model. |
| Lalor & Brown (2016)  Australia * | Cross-sectional | N = 55  n = 29 (f)  n = 27 (m)  Mean age = 10.04 ± 1.39 | BOT-2 | Total sum score of fine and gross motor competence | SPPC: global self-worth subscale | Global self-esteem | Spearman’s rho correlation: The correlation between overall score and global self-worth was not significant. |
| Li (2025)  China | Cross-sectional | N = 452  n = 221 (f)  n = 231 (m)  Mean age 6.14 ± 0.29 | MABC-2: ‘aiming and catching’ and ‘static and dynamic balance’ subscales combined | Total sum score of gross motor competence | SSIS-RS: problem behavior | Total difficulties | Pearson’s bivariate correlation coefficient: There was no correlation between overall gross motor score and problem behaviour (r= -0.28, p<0.01).  Structural equation model: Motor competence negatively associated with problem behaviour (β= -0.23, p<0.05). |
| Lopes (2022)  Portugal | Longitudinal  (24 months) | N = 144  n = 68 (f)  n = 76 (m)  Age range = 4-9 years of age in the beginning | KTK | Total sum score of gross motor competence | PSPP-CY, Portuguese version: global self-esteem subscale | Global self-esteem | Structural equation model: Motor competence significantly predicts global self-esteem in the same year (β=0.20, p=0.036) and one year later (β=0.20, p=0.035). |
| Mancini (2016) Australia | Cross-sectional | N = 93  n = 38 (f)  n = 55 (m)  Mean age = 14.21 ± 1.09 | MABC-2: total score and ‘aiming and catching’ | Total sum score of fine and gross motor competence, and object control skills | MFQ and SCAS | Internalizing problems | Bivariate correlation: There were significant correlations between motor skills and depression (r= -0.33) and anxiety (r= -0.32). Also, object control skills and depression (r= -0.32) and anxiety (r= -0.37) had significant correlations (p<0.001).  Linear multiple regression: Motor skills had a direct effect on depressive symptoms (β= -0.09, 95% BcA CI= -1.67, -0.05) and anxious symptoms (β= -1.27, 95% BcA CI = -2.28, -0.26). |
| Mancini (2018a) Australia | Cross-sectional | N = 164  n = 84 (f)  n = 80 (m)  Mean age = 9.93 ± 1.1 | MABC-2 | Total sum score of fine and gross motor competence | SPPC: global self-worth subscale, and SDQ-P: emotional symptoms, hyperactivity/inattention, and peer problems subscales | Global self-esteem, internalizing problems, and externalizing problems | Bivariate correlation: There were significant (p<0.05) associations from motor skills to peer problems (r= -0.18) and to emotional symptoms (r= -0.29). The association from motor skills to global self-worth (r=0.07) and to attention and hyperactivity difficulties (r= -0.11) weren’t significant.  Mediation analysis: Motor skills demonstrated direct effects to emotional symptoms (β= -0.04, p=0.01) and to peer problems (β= -0.02, p=0.05). |
| Mancini (2018b) Australia | Longitudinal  (18 months), reported cross-sectionally | Time 1 N = 197  n = 95 (f)  n = 102 (m)  Mean age = 5.40 ± 0.30  Time 2 N = 107  n = NR (f)  n = NR (m) | BOT-2SF | Total sum score of fine and gross motor competence | SDQ-T: peer problems subscale, and SSRS | Internalizing problems | Partial correlation: Time 1 Correlations from motor skills to peer problems (r= -0.30) and to internalizing problems (r= -0.17) were significant (p<0.001); Time 2 Correlations from motor skills to peer problems (r= -0.42, p<0.001) and to internalizing problems (r= -0.20, p<0.05) were significant.  Mediation analysis: In time 1 there were no significant direct effect of motor skills on internalizing problems (β= -.001, BcA CI= -0.03,0.03) but it was found on peer problems (β= -.06, BcA CI= -0.09, -0.03); In time 2 there were no significant direct effect of motor skills on internalizing problems (β= 0.02, BcA CI= -0.03, 0.073) but it was found on peer problems (β= -0.082, BcA CI=-0.12, -0.05) as in time 1 model. |
| Noordstar (2020) Netherlands | Cross-sectional | N = 302  n = 152 (f)  n = 150 (m)  Mean age = 9.1 ± 1.6 | The Dutch edition of the MABC-2 | Total sum score of fine and gross motor competence | SPPC, Dutch version: global self-worth subscale | Global self-esteem | Spearman correlation: Motor skills and global self-esteem didn’t have significant correlation (r=0.06). |
| Pullen (2022)  Great Britain | Cross-sectional | N = 224  n = 105 (f)  n = 119 (m)  Mean age = 11.8 ± 1.9 | AIMS and  TJA | Locomotor skills | the Rosenberg Self-Esteem Scale | Global self-esteem | Spearman’s rho correlation: There was no significant correlations found between motor skills and global self-esteem (boys r=0.13, girls r=0.03). |
| Redondo-Tebar (2021) Spain | Cross-sectional | N = 1088  n = 524 (f)  n = 564 (m)  Mean age = 5.32 ± 0.60 | MABC-2: ‘aiming and catching’ and ‘static and dynamic balance’ subscales combined | Total sum score of gross motor competence | Kiddy-KINDL-R, Spanish versio: emotional well-being and self-esteem subscales | Global self-esteem and well-being | Pearson’s correlation: There was significant (p<0.05) correlation found from boys’ motor competence and emotional well-being (r=0.11). Other correlations were not significant: boys’ motor competence and self-esteem (r= -0.00), girls motor competence and emotional well-being (r=0.03), or to self-esteem (r=0.04).  Mediation analysis: The direct association was significant from motor competence to emotional well-being (β=0.14, p<0.05) in boys. Other associations weren’t significant (boys’ motor competence to self-esteem [β= -0.01], girls’ motor competence to emotional well-being [β=0.04], and to self-esteem [β=0.08]. |
| Sortwell (2024) Australia | Cross-sectional | N = 104  n = 56 (f)  n = 48 (m)  Mean age = 9.04 ± 0.69 | CAMSA | Total sum score of gross motor competence | SPPC: global self-worth subscale | Global self-esteem | Pearson’s correlation: There wasn’t significant correlations found from boys’ or from girls’ motor competence and global self-worth (boys r=0.13, girls r=0.03). |
| Tang (2023)  Canada | Cross-sectional | N = 355  n = 166 (f)  n = 183 (m)  n = 6 (non-binary)  Mean age = 7.5 ± 0.5 | PLAYfun | Total sum score of gross motor competence | SDQ-P | Internalizing problems, externalizing problems, and total difficulties | Spearman’s rho correlation: There were significant (p<0.01) correlations found between motor skills and internalizing problems (r= -0.28) and total difficulties (SDQ) (r= -0.19). Between motor skills and externalizing problems, the correlation wasn’t significant (r= -0.07). |
| Abbreviations: f = female, m = male, AIMS = the Athlete Introductory Movement Screen, BOT-2 = the Bruininks–Oseretsky Test of Motor Proficiency Second Edition, BOT-2SF = the Bruininks–Oseretsky Test of Motor Proficiency Second Edition – Short Form, CAMSA = Canadian Agility and Movement Skill Assessment, CES-DC = the 20-item Center for Epidemiologic Studies Depression Scale, KTK = the Körperkoordinationstest für Kinder, PedsQL 4.0 = the Pediatric Quality of Life (QOL) inventory Spanish short form, MABC-2 = the Movement Assessment Battery for Children Second Edition, MFQ = the Mood and Feelings Questionnaire - Child Version, the MOBAK-KG = Kindergarden version, the MOBAK-1-2 = Grades 1-2 version, PSPP-CY = the Physical Self-Perception Profile for Children and Youth, SCAS = the Spence Children’s Anxiety Scale, SPPC = the Self-Perception Profile for Children, SDQ‐P = the Strengths and difficulties questionnaire - parents version, SDQ-T = the Strength and Difficulties Questionnaire Teacher version, SSIS-RS = the Social Skills Improvement System Rating Scale, SSRS = the Social Skills Rating System, TJA = the Tuck Jump Assessment, WEMWBS = the Warwick–Edinburgh Mental Wellbeing Scale, * = Not included in meta-analysis | | | | | | | |

**Supplementary table 3** Risk of bias assessment checklist for studies (NHLBI Study Quality Assessment Tool for Observational Cohort and Cross-Sectional Studies**)**

|  | | 1.  Research question | 2.  Study population | 3. Participation rate of at least 50% | 4. Participant exclusion and inclusion criteria | 5.  Sample size justified | 6. Exposure(s) of interest were measured prior to the outcome(s) being measured | 7.  Sufficient time frame | 8.  Examined different levels of the exposure as related to the outcome | 9.  Clarity of exposure measures | 10.  Was the exposure(s) assessed more than once over time? | 11.  Clarity of dependent variable | 12.  Outcome assessors were blinded | 13.  Loss to follow-up after baseline was 20% or less | 14.  Were key potential confounding variables were addressed | Overall score |  |
| --- | --- | --- | --- | --- | --- | --- | --- | --- | --- | --- | --- | --- | --- | --- | --- | --- | --- |
| Bardid (2016) | | Y | Y | NR | N | Y | N | N | Y | Y | N | Y | NA | NA | N | FAIR |  |
| Bretz (2022) | | Y | Y | Y | Y | N | N | N | Y | Y | N | Y | NA | NA | Y | GOOD |  |
| Chen (2021) | | Y | Y | NR | N | N | N | N | Y | N | N | Y | NA | NA | N | POOR |  |
| de Medeiros (2022) * | | Y | | Y | NR | Y | N | N | N | Y | Y | N | Y | NA | NA | Y | GOOD |
| Gu (2018) | | Y | Y | NR | N | N | Y | Y | Y | Y | N | Y | N | NR | Y | FAIR |  |
| Gu (2019) | | Y | Y | NR | N | N | Y | Y | Y | Y | N | Y | N | NR | Y | FAIR |  |
| Lalor & Brown (2016) * | | Y | Y | NR | NR | N | N | N | Y | Y | N | Y | NA | NA | N | FAIR |  |
| Li (2025) | | Y | Y | NR | Y | N | N | N | Y | Y | N | Y | NA | NA | Y | GOOD |  |
| Lopes (2022) | | Y | Y | NR | Y | N | Y | Y | Y | Y | Y | Y | N | Y | Y | GOOD |  |
| Mancini (2016) | | Y | Y | NR | Y | Y | N | N | Y | Y | N | Y | NA | NA | Y | GOOD |  |
| Mancini (2018a) | | Y | Y | NR | Y | Y | N | N | Y | Y | N | Y | NA | NA | Y | GOOD |  |
| Mancini (2018b) | | Y | Y | NR | Y | Y | N | N | Y | Y | N | Y | NA | NA | Y | GOOD |  |
| Noordstar (2020) | | Y | N | NR | Y | N | N | N | Y | Y | N | Y | NA | NA | N | FAIR |  |
| Pullen (2022) | | Y | Y | NR | N | N | N | N | Y | Y | N | Y | NA | NA | N | FAIR |  |
| Redondo-Tebar (2021) | | Y | Y | N | N | N | N | N | Y | Y | N | Y | NA | NA | Y | GOOD |  |
| Sortwell (2024) | | Y | N | NR | NR | Y | N | N | Y | Y | N | Y | NA | NA | Y | GOOD |  |
| Tang (2023) | | Y | Y | NR | N | Y | N | N | Y | Y | N | Y | NA | NA | Y | GOOD |  |
| Y = yes; N = no; NA = not applicable; NR = not reported; CD = cannot determine. GOOD = low risk of bias; FAIR = some concerns; POOR = High risk of bias. * = Not included in meta-analysis | | | | | | | | | | | | | | | | |  |
